# Restoring metabolic flexibility: targeting organelle interaction networks in the pathogenesis and therapy of MASLD

**DOI:** 10.3389/fcell.2025.1718799

**Published:** 2025-12-03

**Authors:** Yiming Liu, Yue Wang, Jiaying Zhou, Hong Li, Caiyun Liu, Beilei Zhong, Juan Liu, Leiming Liu, Lingling Zhang, Leimin Sun

**Affiliations:** 1 Department of Gastroenterology, The Fourth Affiliated Hospital of School of Medicine, and International School of Medicine, International Institutes of Medicine, Zhejiang University, Yiwu, China; 2 Nursing Department, Sir Run Run Shaw Hospital, Zhejiang University School of Medicine, Hangzhou, China; 3 Key Laboratory of Artificial Organs and Computational Medicine in Zhejiang Province, Institute of Translational Medicine, Zhejiang Shuren University, Hangzhou, China; 4 Center of Metabolic Medicine, International Institutes of Medicine, Zhejiang University, Yiwu, China; 5 Department of Gastroenterology, Sir Run Run Shaw Hospital, Zhejiang University School of Medicine, Hangzhou, China

**Keywords:** metabolic dysfunction-associated steatotic liver disease, metabolic inflexibility, mitochondria, lipid droplets, endoplasmic reticulum stress, autophagy, membrane contact sites, therapeutic targets

## Abstract

Metabolic dysfunction-associated steatotic liver disease (MASLD) is a complex and heterogeneous metabolic disorder where subcellular organelle dysfunction and disrupted inter-organelle communication are recognized as increasingly important drivers of pathogenesis, moving beyond traditional views focused solely on macroscopic metabolic regulation. This review systematically explores the functional impairments of key organelles—including mitochondria, the endoplasmic reticulum, lipid droplets, and autophagic pathways—to delineate their collective roles in fostering lipid metabolism imbalance, oxidative stress, and inflammation. A key innovation discussed is how the pathological dysregulation of membrane contact sites (MCSs) acts as a pivotal mechanism decoupling organelle function and accelerating disease progression. We conclude that therapeutic strategies aimed at restoring cellular metabolic flexibility—by precisely modulating MCSs, activating clearance pathways, and restoring energy metabolism—represent a promising new paradigm for treating MASLD, particularly in patient populations unresponsive to current therapies.

## Graphical Abstract

**Figure:**
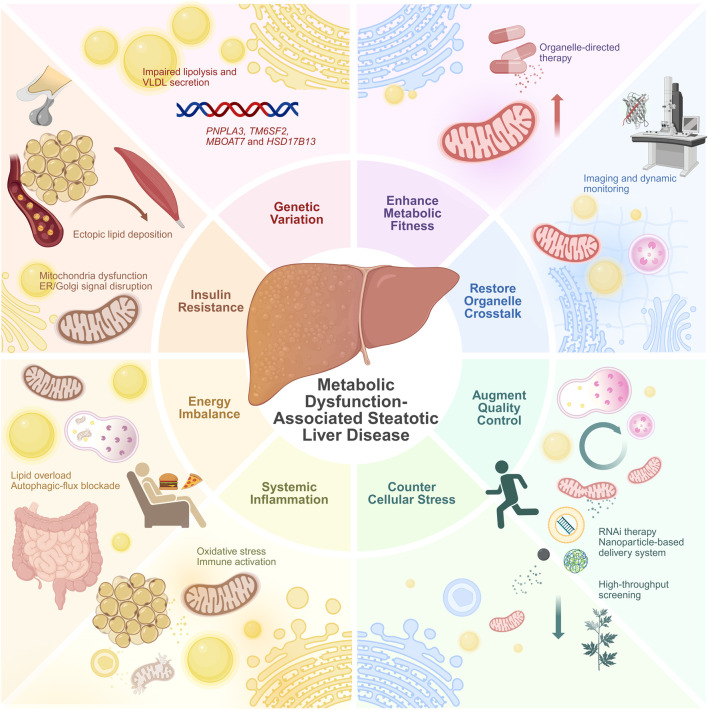

## 1 Introduction

Fatty liver disease is a growing global health problem, but most drugs are still in the research and trial stages. Nevertheless, scientists have made some discoveries about its etiology and pathogenesis, leading to updated nomenclature that replaces the previous exclusion-based term non-alcoholic fatty liver disease (NAFLD). The metabolic dysfunction-associated fatty liver disease (MAFLD) definition proposed in 2020 and the MASLD definition proposed in 2023 both adopt a positive diagnostic approach, requiring both steatosis and metabolic dysfunction (Eslam et al., 2020; Rinella et al., 2023). Both diagnostic criteria incorporate metabolic risk factors, including Body mass index (BMI), waist circumference, blood pressure, blood glucose, and dyslipidemia. Additionally, MAFLD includes elevated high-sensitivity C-reactive protein levels as a risk factor, highlighting the systemic inflammatory state of the disease. Overall, the MAFLD and MASLD criteria have a significant degree of overlap, deepening the impression of the underlying systemic metabolic unhealth and providing a directional guide for drug development (Eslam et al., 2020; Rinella et al., 2023). For the purpose of this review, we will not distinguish between these two concepts and will uniformly refer to them as MASLD.

The MASLD disease spectrum exhibits significant heterogeneity, reflected in patient characteristics (e.g., from lean to obese) and the pace of disease progression. Different mechanistic pathways may exist, linking various combinations of predisposing genes, epigenetic factors, and lifestyles to different disease outcomes. Observable pathological manifestations like hepatic steatosis are at the intersection of these pathways. With the mechanistic pathways not yet fully understood, a mainstream strategy is drug research and trials targeting hepatic and systemic glucose and lipid metabolism disorders. This includes reducing the input of white adipose tissue and blood lipids into the liver, decreasing liver lipid synthesis, and enhancing the liver’s lipid processing and output capabilities. Drug development targeting various pathways has yielded promising results, but challenges remain, including limited applicable populations and adverse reactions. The first approved thyroid hormone receptor beta (THR-β) agonist, resmetirom (Rezdiffra), is indicated for non-cirrhotic non-alcoholic steatohepatitis (NASH) patients with moderate to advanced liver fibrosis (Brisnovali et al., 2024). Multiple clinical trials have shown that the peroxisome proliferator-activated receptor (PPAR)γ agonist pioglitazone and the Glucagon-like peptide-1 (GLP-1) agonist exenatide can improve hepatic steatosis and liver fibrosis, but the main populations included were T2DM patients with NASH (Harrison et al., 2023; Liu et al., 2020). Furthermore, studies found that pioglitazone was significantly more effective in patients with T2DM than in those without (Abdel Monem et al., 2025). Vitamin E shows a better improvement rate in non-diabetic patients, but focuses more on the guidance of dietary trace element intake (Nemer et al., 2024; Song et al., 2025). Among the drugs that have completed Phase 2 clinical trials, the THR-β agonists TERN-501 and TERN-101, the fatty acid synthase (FASN) inhibitor TVB-2640, the Fibroblast growth factor 21 (FGF21) analogs pegozafermin and efruxifermin, the PPAR agonists saroglitazar and lanifibranor, and the GLP-1 agonists efinopegdutide, survodutide, and semaglutide generally enrolled overweight patients or excluded normal-weight patients (Gawrieh et al., 2021; Loomba et al., 2021; Noureddin et al., 2025; Patoulias, 2023; Romero-Gómez et al., 2023). Although the ASK1 inhibitor selonsertib, the PPAR agonist elafibranor, and the C-C chemokine receptor type (CCR) antagonist cenicriviroc did not have strict restrictions on weight and blood glucose, they were halted in Phase 3 trials due to insufficient efficacy (Anstee et al., 2024; Harrison et al., 2020b; Ratziu et al., 2016). In light of the new MASLD definition, a significant proportion of patients not covered by the original criteria lack effective treatments, especially those who are not overweight or have T2DM but have metabolic abnormalities. Previous research has found that these patients often have more severe pathological changes and a tendency to develop hepatitis (Souza et al., 2024).

Although these macro-metabolic regulatory strategies have achieved certain progress, their limitations suggest that deeper pathological mechanisms of MASLD may reside at a more fundamental cellular level. Current pharmaceutical research has paid insufficient attention to the subcellular structures (organelles), due to off-target effects, delivery difficulties, and complex structural functions. However, increasing evidence indicates that the metabolic abnormalities in MASLD are not merely an imbalance between energy intake and output; they also involve a decreased ability of the body to adapt to changes in nutritional status. This phenomenon is known as metabolic inflexibility, where the body cannot efficiently switch between metabolic pathways for substrates like glucose and fatty acids in different states (e.g., fasting and fed), leading to reduced energy utilization efficiency and lipid metabolism disorders (Dukewich et al., 2025). The essence of metabolic inflexibility is failure of the coordinated operation at the subcellular level, manifesting as dysfunction of individual organelles and disruption of their functional cross-talk. For example, under normal conditions, the ER regulates lipid synthesis in the fed state, mitochondria mediate fatty acid oxidation, and lysosomes regulate metabolic balance through autophagy, while lipid droplets (LDs) act as a hub for lipid storage and mobilization. When this organelle coupling is disrupted, metabolic inflexibility worsens, forming a vicious cycle. Therefore, compared to traditional drug interventions targeting systemic metabolic pathways, targeting organelles themselves and their dynamic interactions may provide a new direction for improving metabolic flexibility and halting disease progression. Although this strategy still faces many challenges, advancements in imaging, omics, and targeted delivery technologies are making organelle interaction network-based drug development gradually possible, which will be particularly suitable for the atypical MASLD population that is difficult to cover with traditional therapies.

In summary, MASLD is a highly heterogeneous and mechanistically complex metabolic disease, and its development involves multiple cell types, multi-level metabolic regulation, and critically, organelle dysfunction. In this review, we will focus on the functional changes of major organelles in hepatocytes and related cells in MASLD, explore their key roles in lipid metabolism disorders, oxidative stress, and inflammatory activation, and further elucidate the functional interactions and signaling mechanisms among organelles, with the aim of providing new insights for understanding the pathogenesis of MASLD and exploring potential intervention targets.

## 2 Organellar dysfunction in MASLD

**FIGURE 1:**
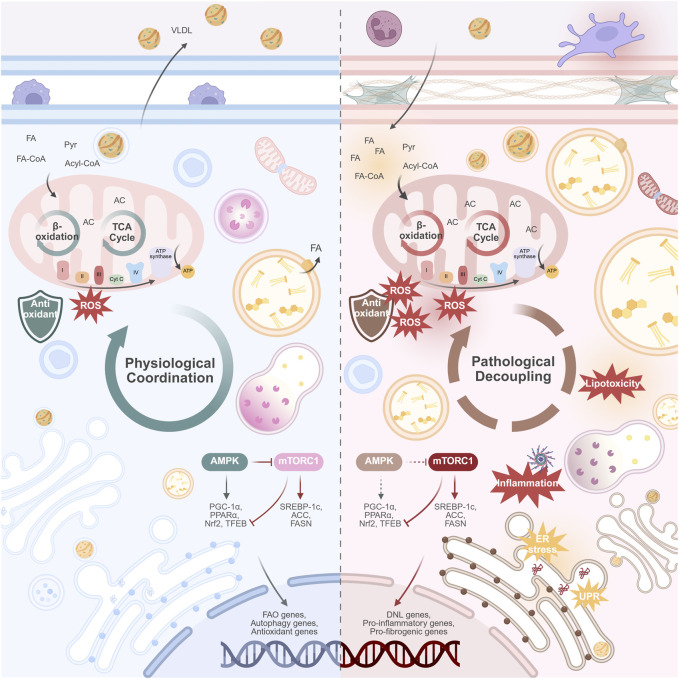
Physiological coordination versus pathological decoupling of organelles in the progression of MASLD. (Left panel) Physiological coordination. In the healthy hepatocyte, organelles work in synchrony. This coordinated state supports metabolic flexibility: efficient mitochondrial β-oxidation and TCA cycle function; balanced lipid storage and lipolysis; robust VLDL secretion; and effective protein handling and quality control. The nutrient-sensing AMPK and mTORC1 pathways are in equilibrium, adapting to energy availability. (Right panel) Pathological decoupling. In MASLD, chronic metabolic stress disrupts organelle communication. This decoupling manifests as mitochondrial dysfunction (impaired β-oxidation, ROS overproduction); ER stress; impaired lysosomal function and blocked autophagic flux; and the formation of enlarged LDs with reduced lipolysis. VLDL secretion is suppressed, contributing to hepatic lipid accumulation. The AMPK/mTORC1 axis is dysregulated, with diminished AMPK activity and persistent mTORC1 activation, promoting lipid synthesis and suppressing catabolic processes. This collective failure drives hepatocyte injury, release of damage-associated molecules, and activation of inflammatory pathways, which ultimately promote hepatic fibrosis. Abbreviations: ACC, acetyl-CoA carboxylase; AC, Acyl-CoA; AMPK, AMP-activated protein kinase; DNL, *de novo* lipo-genesis; ER, endoplasmic reticulum; FA, fatty acid; FA-CoA, Fatty acyl-CoA; FAO, fatty acid oxidation; FASN, fatty acid synthase; MASLD, metabolic dysfunction-associated steatotic liver disease; mTORC1, mechanistic target of rapamycin complex 1; Nrf2, nuclear factor erythroid 2-related factor 2; PGC-1α, peroxisome proliferator-activated receptor gamma coactivator 1-alpha; PPARα, peroxisome proliferator-activated receptor alpha; Pyr, Pyruvate; ROS, reactive oxygen species; SREBP-1c, sterol regulatory element-binding protein 1c; TCA, tricarboxylic acid cycle; TFEB, transcription factor EB; UPR, unfolded protein response. Created in BioRender.

Recent studies have found that multiple organelles exhibit functional abnormalities and structural disorganization during MASLD progression, covering several key pathways from energy metabolism imbalance to exacerbated oxidative stress and impaired autophagy. These abnormalities include both early adaptive responses to lipid overload and functional failure in the decompensated state, reflecting the “dynamic fragility” of organelle regulatory capacity. Although observations of these changes vary among different studies, the overall trend suggests that organelle dysfunction may play an important role in the development of MASLD. This section will review the functional changes and potential pathological significance of major organelles in MASLD, focusing on key mechanisms such as energy metabolism disorders, abnormal stress responses, and autophagy imbalance (Figure 1).

### 2.1 Energy metabolism disorder

Energy metabolism disorder is one of the core mechanisms of MASLD, spanning multiple stages from simple steatosis to inflammation and fibrosis. As one of the cell types with the highest metabolic load, hepatocytes rely on the coordinated cooperation between mitochondria and LDs to achieve a dynamic balance of substrate mobilization, oxidation, and energy conversion.

In early MASLD patients, hepatocytes compensate for the lipid load by upregulating mitochondrial oxidative phosphorylation, β-oxidation, and the tricarboxylic acid cycle (TCA) activity. Their TCA activity is positively correlated with intrahepatic TAG levels (Koliaki et al., 2015; Sunny et al., 2011). However, this compensation is not long-lasting. Continuous lipid accumulation leads to mitochondrial dysfunction: the activity of the electron transport chain (ETC.) complexes (especially I and III) decreases (Moore et al., 2022; Pedersen et al., 2022). Meanwhile, in patients and high-fat diet (HFD) mouse models, as another adaptive (or stress) response to lipid overload, the activity of mitochondrial Cytochrome P450 (CYP)2E1 increases (Ofosu-Boateng et al., 2024; Wei et al., 2023). These two factors jointly lead to a dramatic increase in reactive oxygen species (ROS) production, which exceeds the antioxidant clearance capacity, causing mitochondrial damage and the release of molecules like mitochondrial DNA (mtDNA), which activate downstream inflammatory pathways (e.g., cGAS-STING). Continuous ROS accumulation, mitochondrial damage, and inflammatory response ultimately drive hepatocyte damage, apoptosis, liver fibrosis, and even hepatocellular carcinoma (HCC).

As a key synergistic organelle for mitochondrial energy metabolism, lipid droplet (LDs) dysfunction is another important driver of energy homeostasis imbalance in MASLD. In MASLD patients and HFD mouse models, the cholesterol content within LDs increases, leading to elevated lipid droplet viscosity (Li et al., 2026; Sakuma et al., 2025). Downregulation of adipose triglyceride lipase (ATGL), the rate-limiting enzyme for lipolysis, and concurrent overexpression of LDs-associated proteins, including Perilipin 1 (PLIN1), Perilipin 2/3 (PLIN2/3), and Cell Death-Inducing DFFA-Like Effector B (CIDEB), collectively promote the formation of enlarged LDs. The Perilipins (PLINs) are essential structural components that coat LDs, while CIDEB is known to mediate LD fusion (He et al., 2022; Zhang et al., 2025). This process restricts the efficient supply of fatty acids while simultaneously occupying cellular space, and even compresses the space of Disse to affect hepatic sinusoidal microcirculation. Recent evidence indicates that, during the progression of MASLD, peridroplet mitochondria (PDM) become increasingly abundant, whereas cytoplasmic mitochondria (CM) are reduced. PDM are characterized by higher pyruvate oxidation capacity and respiratory activity; however, with disease progression, they exhibit impaired fatty acid oxidation (FAO) together with enhanced *de novo* lipogenesis (DNL), thereby favoring lipid droplet expansion, as demonstrated in a choline-deficient, high-fat diet (CDAHFD) mouse model (Talari et al., 2025). Such an imbalance between mitochondrial subpopulations further aggravates disturbances in cellular energy metabolism.

Overall, during MASLD progression, the decoupling between mitochondria and LDs in hepatocytes disrupts the metabolic linkage between lipid mobilization and energy conversion, exacerbating metabolic inflexibility, and ultimately leading to a series of pathological changes such as lipid deposition, energy disorders, and cell damage.

### 2.2 Metabolic sensing and stress signaling imbalance

#### 2.2.1 Energy sensing disorders and metabolic inflexibility

In a healthy state, hepatocytes sense nutritional status through a complex signaling network and rely on the functional coordination of organelles like mitochondria and lysosomes to dynamically regulate energy metabolism. As core energy sensors, the AMPK and mTORC1 pathways are antagonistic. AMPK is activated when energy is scarce (mainly sensing the increase in the mitochondrial AMP/ATP ratio). It not only promotes macro glucose and lipid metabolism but also phosphorylates ULK1 to inhibit mTORC1, promoting the formation and fusion of autophagosomes, while also activating proliferator-activated receptor gamma coactivator 1-alpha (PGC1α)-mediated mitochondrial biogenesis and BNIP3–LC3-mediated mitophagy to improve mitochondrial membrane potential and ROS clearance efficiency. Meanwhile, mTORC1 (localized to the lysosomal membrane) is activated when nutrients are abundant, promoting lipid synthesis and cell growth. A delicate bidirectional inhibitory balance exists between the two (Sadria and Layton, 2021).

In MASLD, impaired mitochondrial oxidative phosphorylation leads to a decrease in ATP production, which should theoretically activate AMPK. However, studies have found that AMPK activity is paradoxically and persistently inhibited (related to enhanced inhibitory phosphorylation at Ser485, as shown in HFD mouse model, and decreased activity of the upstream kinase LKB1, as shown in *in vitro* hepatic steatosis model) (Pan et al., 2024; Wang et al., 2021). This leads to reduced fatty acid oxidation and autophagy. At the same time, mTORC1 exhibits pathological persistent activation, promoting lipid synthesis and inhibiting cell renewal (Palomer et al., 2025; Tohamy et al., 2025).

The long-term AMPK-mTORC1 signaling imbalance has profound consequences: it not only weakens the cell’s ability to respond to changes in energy status but also exacerbates mitochondrial dysfunction, inhibits autophagy, and promotes lipid droplet accumulation. This forms a self-reinforcing pathological cycle at the “nutrient sensing–organelle interaction–metabolic regulation” level, laying the foundation for subsequent cellular stress, damage, and even death.

#### 2.2.2 Dysregulation of stress compensation and organelle instability

When metabolic load continues to increase, hepatocytes rely on organelle stress responses to maintain homeostasis. However, in MASLD, these responses are often from adaptive compensation to uncontrolled activation, leading to organelle functional instability.

##### 2.2.2.1 ER stress and calcium signaling dysregulation

Toxic lipids, such as saturated fatty acids, and ROS induce endoplasmic reticulum (ER) stress, activating the three branches of the unfolded protein response (UPR). The activated IRE1α RNase domain splices XBP1 mRNA to induce ER-associated degradation (ERAD)-related gene expression. Simultaneously, hyperactivation of the RIDD pathway leads to the degradation of non-essential mRNAs and miRNAs, ultimately triggering apoptosis. The PERK pathway is also persistently activated, which in turn phosphorylates eukaryotic initiation factor 2α (eIF2α) to initiate the integrated stress response (ISR), thereby suppressing global protein synthesis (Ajoolabady et al., 2023). Elevated UPR markers are significantly observed in MASLD patients’ liver tissue, confirming a state of persistent and pathological UPR activation (González-Rodríguez et al., 2014). HFD mouse model and *in vitro* studies revealed that uncontrolled ER stress disrupts the integrity of ER-mitochondria membrane contact sites (MCSs) by compromising key molecules (e.g., PACS-2; Mitofusin2, MFN2). This disruption leads to abnormal InsP3R channel function and increased calcium ion release from the ER. Together with depleted ER calcium stores and inhibited SERCA activity, this cascade culminates in mitochondrial calcium overload, further driving ROS bursts and cellular damage (Jiang et al., 2025; Ryu et al., 2024; Zhao and Sheng, 2024).

##### 2.2.2.2 Integrated stress response and downstream effects

Phosphorylation of eIF2α serves as a central event in ISR, which initiates cellular adaptive programs by globally suppressing protein synthesis while selectively promoting the translation of activating transcription factor 4 (ATF4). ATF4 subsequently coordinates the upregulation of stress-responsive gene networks, including C/EBP homologous protein (CHOP) (Shpilka and Haynes, 2018). Study in HFD mouse model has demonstrated persistent activation of the PERK–eIF2α–ATF4 axis during MASLD progression, accompanied by an initial increase followed by a decline in mitochondrial UPR components (e.g., LONP1) and mitokines (e.g., GDF15, FGF21), paralleled by worsening mitochondrial dysfunction (Yuan et al., 2025). As a downstream mitokine, FGF21 partially alleviates hepatic steatosis and stress through a negative feedback mechanism (H. Wei et al., 2025). Therefore, the ISR dynamically links organelle function in MASLD, making the restoration of its declining protective components, such as mitokines, a promising therapeutic strategy.

##### 2.2.2.3 Mitochondrial stress and inflammatory signal amplification

Mitochondrial dysfunction manifests as a dramatic increase in ROS, a decrease in membrane potential, and mtDNA damage. In MASLD patients and in mouse models fed methionine- and choline-deficient (MCD) or HFD, damaged mitochondria release various signaling molecules that act as Danger-Associated Molecular Patterns (DAMPs), initiating and amplifying inflammatory signals, such as the cGAS-STING pathway, the NLRP3 inflammasome, and the NF-κB pathway. These signals, in concert with other stimuli, directly or indirectly trigger the activation of hepatic stellate cells (HSCs) (An et al., 2020; Giacco et al., 2024; Yu et al., 2019), which drives the progression of liver fibrosis.

##### 2.2.2.4 Autophagy impairment and stress dysregulation

The overall impairment of autophagic flux is a key amplifier of organelle instability and stress dysregulation in MASLD. Blocked protective autophagy induced by the UPR pathway exacerbates the persistent activation of ER stress. The IRE1α-XBP1 and PERK-eIF2α-ATF4 arms of the UPR promote autophagy as an adaptive response to remove unfolded proteins and damaged organelles (Hetz et al., 2020). In MASLD patients and in MCD or HFD mouse models, under persistent stress, however, this cytoprotective autophagy is disrupted by pathways including mTOR, resulting in accumulated misfolded proteins. Ultimately, through the actions of CHOP and JNK, the cellular response shifts from adaptive to pro-apoptotic, amplifying ER stress and activating cell death pathways (González-Rodríguez et al., 2014). Blocked mitophagy leads to the accumulation of damaged mitochondria, which promotes mtDNA leakage and the production of toxic lipids, accelerating the cascade of damage.

In summary, persistent metabolic disorders in MASLD activate multiple stress pathways, but due to organelle dysfunction, imbalanced signal regulation, and impaired autophagy capacity, compensatory stress responses turn into persistent damage signals. Uncontrolled stress responses continuously amplify cell damage, driving disease progression toward fibrosis, cirrhosis, and HCC.

### 2.3 Autophagy and organelle quality control imbalance

The autophagy system is a core mechanism for maintaining organelle quality homeostasis, ensuring metabolic balance by selectively clearing damaged organelles (e.g., mitochondria, ER, LDs). In MASLD, impaired autophagy leads to a blockage of the “clearance-renewal” process, resulting in the accumulation of damaged organelles and harmful substances, which further amplify stress responses and inflammatory signals.

The significant accumulation of the autophagy substrate p62/SQSTM1 in liver tissue from MCD or HFD mouse models suggest impaired autophagic flux (Ding et al., 2020). MASLD animal models and *in vitro* studies further revealed the specific mechanisms, include: excessive activation of mTORC1 and inhibition of AMPK leading to blocked autophagy initiation; downregulation of Vps34, Beclin-1, and other Atg proteins affecting the nucleation and elongation of autophagosomes (J. Li et al., 2025; Yang et al., 2024); defective SNARE complex formation impairing autophagosome-lysosome fusion (Rho et al., 2024); and intrinsic lysosomal dysfunction, including acidification defects (impaired V-ATPase function) and reduced activity of lysosomal acid lipase (Sun et al., 2025; Zeng et al., 2023).

Mitophagy is reduced in MASLD, and mitochondria show ultrastructural abnormalities such as fragmentation and cristae rupture, accompanied by Mitofusin (MFN) downregulation and Dynamin-related protein (Drp)1 regulatory imbalance, both in MASLD patients and HFD mouse models, suggesting abnormal mitochondrial dynamics (Bórquez et al., 2024; Padelli et al., 2025; Zhang et al., 2025). The activation of key mitophagy pathways (e.g., PINK1/Parkin or BNIP3/NIX receptor pathways) is impaired, leading to the ineffective clearance of damaged mitochondria, which further activate inflammatory pathways (K. He et al., 2025; Wang et al., 2021; Zhang et al., 2019).

Lipophagy is also inhibited. In high-fat and high-fructose diet (HFFD) rat models and MASLD patients, LC3 and Rab7, which mediate lipid droplet macroautophagy, and LAMP2A, a Chaperone-mediated autophagy (CMA)-related protein that mediates the contact between LDs and lysosomes, are downregulated, suggesting impaired lipophagy. CMA can promote macroautophagy by reducing the size of LDs. Impairment of both lipolysis and lipophagy exacerbates the formation of giant LDs. In liver grafts, giant LDs are associated with more severe post-transplant fibrosis, as they may increase susceptibility to ischemia-reperfusion injury (Ferri et al., 2021; Baidya et al., 2020).

While autophagy is a recognized protective mechanism in MASLD, its role in certain cell types is more complex. For example, in HSCs, study from MASLD patients and MCD mouse revealed autophagy functions as a protective mechanism by degrading LDs and other organelles to inhibit HSC activation and fibrosis; yet, it has also been found to pathologically promote HSC survival and proliferation in another *in vitro* study, thereby driving hepatic fibrosis (Cao et al., 2022; Duan et al., 2017). Therefore, the role of autophagy in MASLD warrants further investigation.

### 2.4 Biogenesis and material transport disorder

Ribosomes, the ER, and the Golgi apparatus constitute the synthesis-transport network of hepatocytes. The abnormalities of these organelles lead to impaired lipoprotein assembly and secretion, and unbalanced protein synthesis and transport, which exacerbate hepatic lipid accumulation and metabolic disorders in MASLD.

Protein synthesis is aberrantly regulated in MASLD. Activation of the ER UPR branch PERK-eIF2α-ATF4 promotes the expression of stress-related genes while inhibiting global protein translation (Ajoolabady et al., 2023). Furthermore, studies have shown that the expression of ribosomal proteins (e.g., rpS6, rpL3, RACK1) and several eukaryotic initiation factors (e.g., eIF1, eIF3a) is downregulated in MASLD patients, indicating a general decline in the efficiency of ribosomal translation initiation. In contrast, eIF6 expression is specifically upregulated, and this unique upregulation impairs mitochondrial function and may promote disease progression toward HCC (Scagliola et al., 2021). This suggests that translation in MASLD exhibits a complex and selective dysregulation.

Lipid synthesis and transport are also aberrantly regulated in MASLD, in which the Golgi apparatus plays a critical role. PNPLA3-I148M, a known genetic determinant of MASLD, can affect Golgi structure by increasing Golgi cisternal width and promoting its contact with LD (Sherman et al., 2024). In MASLD, the accelerated degradation of transport proteins SEC22B and STX5A—located on the membranes of ER-derived vesicles and the Golgi—reduces the efficiency of very-low-density lipoprotein (VLDL) transport from the ER to the Golgi (Zhang et al., 2025). Additionally, abnormal elevation of the Golgi protein 73 has been observed in lean MASLD patients, which compromises VLDL assembly and secretion by inhibiting ApoB100 (Peng et al., 2021). On the other hand, the CD36-regulated transport and processing of SREBP1 from the ER to the Golgi are increased in HFD mouse models, which further promotes DNL (Zeng et al., 2022). Therefore, Golgi transport dysfunction simultaneously impairs lipoprotein secretion while exacerbating DNL.

### 2.5 Detoxification and antioxidant disorder

The progression of MASLD is characterized by a dynamic imbalance in hepatocyte antioxidant and detoxification systems, marked by an “overload-compensation-exhaustion” cycle.

Mitochondria, under conditions of excessive β-oxidation, become a central driver of oxidative stress through the leakage of electrons from the electron transport chain and the subsequent generation of ROS. Peroxisomes, as another site for β-oxidation, directly produce hydrogen peroxide (H_2_O_2_) as a byproduct. Under physiological conditions, low levels of ROS are promptly scavenged by catalase (CAT) and also act as signaling molecules that activate the peroxisomal membrane protein PEX2. This leads to the post-translational modification of ATGL on LDs, promoting its degradation and thereby moderately inhibiting lipolysis to prevent excessive accumulation of fatty acids and ROS. In addition, recent research in human cell models indicates that peroxisomes can form physical contacts with mitochondria, facilitating the transfer of ROS to help maintain mitochondrial health (DiGiovanni et al., 2025). In MASLD, the downregulation of PPARα signaling leads to reduced peroxisome biogenesis and suppressed CAT activity, resulting in the massive accumulation of H_2_O_2_ and lipid peroxides. Building upon this, the expression of CYP2E1 and CYP4A located in the ER and mitochondria-associated ER membranes (MAMs) is upregulated, as shown in MASLD patients and multicellular organotypic liver model. This leads to the synergistic production of excessive ROS through electron leakage and collaboration with NADPH oxidase (NOX), accompanied by the generation of toxic lipids, thereby exacerbating localized oxidative damage (Ryu et al., 2019; Wang and Liu, 2025; Wei et al., 2023). The consequent elevated ROS levels hyperactivate PEX2, increasing ATGL degradation and further aggravating hepatic lipid accumulation in another *in vitro* study (Ding et al., 2021).

As the disease progresses, the antioxidant defense system of MASLD hepatocytes gradually becomes depleted. Evidence from a study using HFD mouse models demonstrates that antioxidant molecules such as glutathione (GSH), superoxide dismutase (SOD), and CAT are significantly reduced in the later stages, which may be associated with decreased activity of the mitochondrial Nrf2-Keap1 pathway (Zhang et al., 2025). Simultaneously, the organelle-associated detoxification activity, which uses GSH as a substrate, also declines. This prevents the conversion or excretion of toxic metabolites, leading to their accumulation in organelles like the ER and further aggravating their structural and functional damage (Svobodová et al., 2025).

## 3 Membrane contact sites and MASLD

**FIGURE 2:**
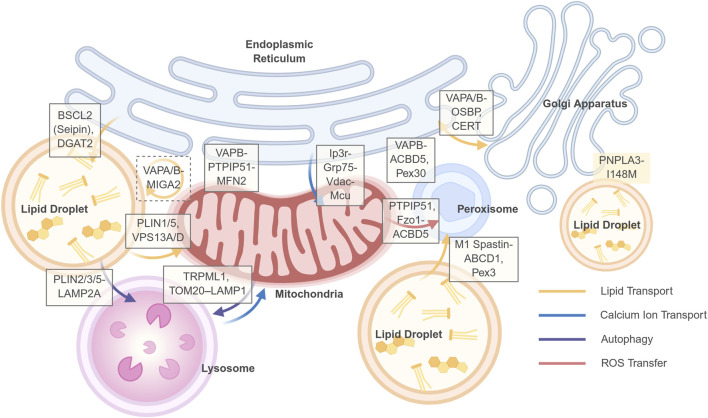
Key membrane contact sites (MCSs) and their functions in hepatocyte organelle communication. This schematic illustrates a network of select membrane contact sites (MCSs) between organelles that are critical for maintaining hepatic metabolic homeostasis. The tethering proteins or protein complexes that form and regulate these contact sites are indicated in white boxes positioned at the respective organelle interfaces: Solid line frame: two-way contacts; Dashed line frame: three-way contacts. No border: proteins associated with contact site regulation. The dynamic exchange of metabolites, ions, and signals across these MCSs is represented by colored arrows, as defined in the legend in the lower right corner: Yellow arrows: Lipid transport; Blue arrows: Calcium ion (Ca^2+^) transport; Purple arrows: Autophagic processes; Red arrows: Reactive oxygen species (ROS) transfer. Created in BioRender.

Hepatocytic metabolic flexibility relies on the highly coordinated functionality of organelles. Inter-organelle communication encompasses the diffusion of cytosolic signaling molecules, vesicular trafficking (e.g., VLDL secretion and autophagic flux, as mentioned previously), and MCSs. Among these, MCSs—tethered by protein complexes and maintaining an intermembrane space of approximately 10–30 nm—represent a rapid and precise form of non-vesicular communication (Calì et al., 2025). Dysfunction of MCSs, leading to imbalances in the direct exchange of lipids, ions, and metabolites, is a key mechanism underpinning the decoupling of organelle interactions (Figure 2).

In early MASLD mouse models, increased lipid load induces a compensatory upregulation of MCSs, manifested by MAMs expansion and an increase in PDM (Talari et al., 2025). This adaptation contributes to mitochondrial dynamics and tight coupling with the endoplasmic reticulum and lipid droplets, facilitating fatty acid trafficking and disposal, enhances calcium homeostasis, and boosts energy supply (Friedman et al., 2011; Henne, 2016). However, as lipid accumulation persists and surpasses metabolic and storage capacities, MCSs become aberrant (Table 1). For instance, evidence from both *in vivo* and *in vitro* experiments demonstrates that, complexes localized to MAMs, such as the IP_3_R-Grp75-VDAC axis, mediate excessive calcium transfer, resulting in mitochondrial calcium overload and ROS burst (Lai et al., 2025). This process is frequently exacerbated by the altered expression or function of MFN2, a well-established tethering and regulatory protein at the MAMs (Bórquez et al., 2024; Naón et al., 2023; Sun et al., 2025). Concurrently, dysregulation of key proteins at ER-LD contact sites, such as Seipin, promotes the generation of excessive LDs, providing a source of lipotoxicity. Furthermore, dysfunction in mitochondria-LD and lysosome-LD contacts impairs the efficient degradation of LDs. These MCSs’ functions are not isolated but constitute a dynamic network. Consequently, targeting the dynamics of MCSs presents a novel therapeutic strategy. Notably, the architecture of the MCSs network is not stochastic but exhibits high efficiency, whereby a limited set of universal adaptor molecules are recurrently employed to mediate diverse membrane contact events. Key nodes include VAPA/B on the ER, the ORP/OSBP family, Rab7 on lysosomes, and the VPS13 family, which facilitates bulk lipid transport. Targeting these central hubs may therefore yield broader therapeutic benefits by coordinately regulating inter-organelle communication. Current advances in highly sensitive tools—including genetically encoded probes based on split fluorescent proteins (e.g., SPLICS), FRET/BRET, and proximity labeling techniques (e.g., BioID, APEX)—enable the visualization of MCSs’ dynamics and the identification of their molecular composition in living cells and even animal models, providing robust support for target validation (Calì et al., 2025).

**TABLE 1:** Overview of membrane contact site (MCS) dysfunction in metabolic dysfunction-associated steatotic liver disease (MASLD).

MCS contact pair	Core function in MASLD	Key proteins/molecules	Dynamic changes in MASLD	Disease model evidence	Refs
ER - Lipid Droplet (ER-LD)	LD biogenesis, TG synthesis and deposition	BSCL2 (Seipin), DGAT2	Enhanced/Hyperactive: HFD upregulates DGAT2 expression, catalyzing TG synthesis, leading to aberrant LD expansion and maturation impairment	Phase II clinical trials confirm DGAT2 inhibition (ION224) significantly improves liver steatosis and histology in MASH patients. Animal models show DGAT2 inhibition increases ER phosphatidylethanolamine (PE) levels, suppressing SREBP-1c cleavage and lipogenesis. Cellular models indicate Seipin loss reduces cholesterol-mediated large LD formation and alleviates ER stress	Demirel-Yalciner et al. (2024), Loomba et al. (2025), Ouyang et al. (2024), Rong et al. (2024)
ER - Mitochondria (ER-Mito)	Phospholipid (PS) transfer, Ca^2+^ signaling, mitochondrial function and dynamics	VAPB, PTPIP51, MFN2, IP3R, GRP75, VDAC	Biphasic Dysregulation: Early contact enhancement causes Ca^2+^ overload and lipotoxicity; late contact disruption impedes PS transfer and β-oxidation. Both increased and decreased contact promote lipid accumulation	MFN2 is downregulated in livers of MASLD patients. Impaired IP3R-GRP75-VDAC/MCU-mediated PS transfer is observed in HFD-fed mouse and yellow catfish models. Upregulating Mfn2 in HFD mouse models restores MAMs and improves lipid metabolism. Imbalanced Mfn2/Grp75 expression causes lipid accumulation via different mechanisms (ER stress or mitochondrial dysfunction)	Bassot et al. (2021), Jiang et al. (2025), Lai et al. (2025)
Mitochondria - LD (Mito-LD)	Fatty acid oxidation and lipolysis, mitochondrial function	PLIN1/5, VPS13A/D	Enhanced but dysfunctional: HFD drives increased contact to promote substrate flux, but aberrant PLIN expression inhibits lipolysis, reducing FA transfer efficiency from LD to mitochondria	Animal models (mice) show Plin5 knockout ameliorates hepatic steatosis and HCC development; RIPK3 deficiency upregulates PLIN1/5, improving mitochondrial function and LD dynamics. HFD induces VPS13A/D relocation from mitochondria to LDs, mediating contact	Afonso et al. (2023), Krahmer et al. (2018), Mass-Sanchez et al. (2024)
Three-way: ER-Mito-LD	Coordinates lipid flow, efficiently channels mitochondrial FA precursors to ER for TG synthesis and storage in LDs	VAPA/B, MIGA2	Likely enhanced/dysfunctional: As an adaptive response, HFD may drive this hub formation to handle lipid overload; its dysfunction may lead to inefficient lipid flow	MIGA2 overexpression promotes LD formation in adipocytes. Its function depends on binding VAPA/B. Structural studies reveal its phosphatidylserine (PS) transport capability. Its physio-pathological role in hepatic lipid metabolism requires further investigation	Freyre et al. (2019), Kim et al. (2022)
ER - Golgi	Cholesterol/sphingolipid metabolism and trafficking	OSBP, CERT, VAPA/B	Enhanced: Drives synthesis and trafficking of lipotoxic sphingolipids like ceramide	Animal models reveal TTC39B influences hepatic lipogenesis via VAPB/SCAP interaction; the SphK2-CERT axis promotes ceramide conversion to sphingomyelin (SM), driving NAFLD-HCC progression. *In vitro* reconstitution studies confirm ORP/OSBP sterol transport relies on PI4P gradients. Its core role in hepatic lipogenesis awaits further validation in animal models	Fuggetta et al. (2024), Hsieh et al. (2022), Liu et al. (2022)
Lysosome - LD (Lys-LD)	Lipophagy	Rab7, PLIN2/3/5, LAMP2A	Attenuated: Impaired autophagic flux reduces lysosomal LD degradation efficiency	Rab7 is identified as a key activator of starvation-induced lipophagy in primary mouse hepatocytes; its regulation in overnutrition models (e.g., HFD) remains unclear. Acute lipid overload mouse models and *ex vivo* human liver tissue reveal mTORC1 initiates lipophagy by phosphorylating Plin3. MASLD patient livers show reduced LAMP2A, Plin5 accumulation, and impaired CMA function	Garcia-Macia et al. (2021), Ma et al. (2020), Schroeder et al. (2015)
Mitochondria - Lysosome (Mito-Lys)	Mitophagy, mitochondrial dynamics, Ca^2+^ crosstalk	Rab7, TBC1D15, FIS1, TRPML1	Likely attenuated/dysfunctional: Possibly impaired by lipotoxicity, compromising mitochondrial quality control	Cellular studies reveal Rab7/TBC1D15/Fis1 and TRPML1 mediate direct Mito-Lys contact, regulating fission and Ca^2+^ signaling. Mitophagy is downregulated in steatotic human livers, but direct changes in Mito-Lys contact remain unknown	Peng et al. (2020), Wang et al. (2021), Wong et al. (2018)
ER - Peroxisome (ER-Pex)	VLCFA metabolism; Peroxisome biogenesis	ACBD5, VAPB, Pex30	Likely enhanced (compensatory): May increase in response to lipid overload, but likely insufficient to counteract overall lipid accumulation	Cellular studies show ACBD5 depletion reduces ER-peroxisome contacts, causing VLCFA accumulation. In yeast, Pex30 maintains ER-peroxisome integrity and phosphatidic acid (PA) metabolism. PKC signaling positively regulates ACBD5-VAPB and promotes biogenesis. Its dynamics and function in animal livers and MASLD require direct confirmation	Borisyuk et al. (2025), Costello et al. (2023), Ferreira et al. (2025)
Mitochondria - Peroxisome (Mito-Pex)	β-oxidation collaboration; Mitochondrial fusion; Mitochondrial antioxidant defense	ACBD5, PTPIP51, Fzo1/MFN2	Likely enhanced/dysfunctional: May increase contact responding to lipid overload, but inefficient metabolite (e.g., citrate) or ROS exchange fails to alleviate metabolic stress	In yeast, Fzo1 (MFN2 homolog) mediates contact, promoting FA β-oxidation and citrate transfer to sustain mitochondrial fusion. Huh7 cell studies show ACBD5-PTPIP51 mediates contact, increasing during oxidative stress for ROS transfer. Its alteration and function in MASLD is an emerging research focus	Alsayyah et al. (2024), DiGiovanni et al. (2025), Shai et al. (2018)
Peroxisome - LD (Pex-LD)	β-oxidation, Lipolysis	Spastin, ABCD1, Pex3, Tgl4	Likely enhanced (compensatory): Contact may increase to promote FA shunting and oxidation, but function may be insufficient	M1 Spastin-ABCD1 recruits ESCRT-III proteins to promote FA trafficking from LDs to peroxisomes. The herbal formula JTTZF ameliorates hepatic steatosis and oxidative stress in HFD-induced MASLD mice by targeting the Pex-LD interface (e.g., downregulating ABCD2, an ABCD1 homolog)	Amado et al. (2025), Chang et al. (2019), Miao et al. (2025)
Three-way: ER-Pex-LD	Coordinates cooperative biogenesis of LDs and peroxisomes	Pex30/MCTP2	Unknown	Evolutionary conserved studies (yeast/plants) show LDs and peroxisomes co-emerge from shared ER subdomains (e.g., Pex30 domains). Its pathophysiological significance in mammalian liver lipid metabolism remains a frontier	Joshi et al. (2018), Wright et al. (2025)

Abbreviations: ABCD1/2, ATP-Binding Cassette Sub-family D Member 1/2; ACBD5, Acyl-CoA, Binding Domain Containing 5; BSCL2, Berardinelli-Seip Congenital Lipodystrophy 2; CERT, ceramide transfer protein; CMA, Chaperone-Mediated Autophagy; DGAT2, Diacylglycerol O-Acyltransferase 2; ER, endoplasmic reticulum; ESCRT-III, Endosomal Sorting Complex Required for Transport-III; FA, fatty acid; FIS1, fission, Mitochondrial 1; GRP75, Glucose-Regulated Protein 75; HCC, hepatocellular carcinoma; HFD, High-Fat Diet; IP3R, Inositol 1,4,5-Trisphosphate Receptor; JTTZF, Jiang-Tang-Tiao-Zhi Formula; LAMP2A, Lysosomal Associated Membrane Protein 2A; LD, lipid droplet; MAMs, Mitochondria-Associated ER, membranes; MASLD, Metabolic Dysfunction-Associated Steatotic Liver Disease; MASH, Metabolic Dysfunction-Associated Steatohepatitis; MCU, mitochondrial calcium uniporter; MCTP2, Multiple C2 and Transmembrane Domain Containing 2; MFN2, Mitofusin 2; MIGA2, Mitoguardin 2; mTORC1, Mechanistic Target of Rapamycin Complex 1; NAFLD, Non-Alcoholic Fatty Liver Disease; OSBP, Oxysterol-Binding Protein; PA, phosphatidic acid; PE, phosphatidylethanolamine; Pex3/30, Peroxisomal Biogenesis Factor 3/30; PKC, Protein Kinase C; PLIN1/2/3/5, Perilipin 1/2/3/5; PS, phosphatidylserine; PTPIP51, Protein Tyrosine Phosphatase Interacting Protein 51; Rab7, Ras-related protein Rab-7; RIPK3, Receptor-Interacting Serine/Threonine-Protein Kinase 3; ROS, reactive oxygen species; SCAP, SREBP, Cleavage-Activating Protein; SM, sphingomyelin; SphK2, Sphingosine Kinase 2; SREBP-1c, Sterol Regulatory Element-Binding Protein 1c; TBC1D15, TBC1 Domain Family Member 15; TG, triglyceride; Tgl4, Triglyceride Lipase 4; TRPML1, transient receptor potential cation channel, Mucolipin Subfamily, Member 1; TTC39B, Tetratricopeptide Repeat Protein 39B; VAPA/B, VAMP-Associated Protein A/B; VDAC, Voltage-Dependent Anion Channel; VLCFA, Very Long-Chain Fatty Acid; VPS13A/D, Vacuolar Protein Sorting-Associated Protein 13A/D.

## 4 Current status of organelle-targeting drug development and potential targets

In recent years, although drugs like resmetirom, pioglitazone, and exenatide have shown efficacy in the MASLD field, MASLD treatment is still plagued by issues such as limited patient populations, varying efficacy, and safety concerns. Among the numerous mechanisms, clinical trials often emphasize the macroscopic effects of these drugs on “weight loss, improved insulin sensitivity, and reduced liver fat”, while attention to their effects at the organelle level is often lagging and comes from animal or *in vitro* models. This structure reflects a hierarchy of evidence from the macro to the micro, from clinical to basic research, but it also means we may have overlooked the potential efficacy of these drugs in specific patients (e.g., lean MASLD) and underestimated the potential of these drugs for precise treatment by targeting unique pathological defects through organelle function compensation (Table 2).

**TABLE 2:** Summary of the mechanism of action and organelle targets of representative drugs for the treatment of MASLD.

Drug name	Stage	Mechanism of action	Effect on organelles/organelle interactions	Primary organelle/organelle interaction targeted
Rezdiffra (Resmetirom)	FDA Approved (for MASH with fibrosis)	THR-β agonist. Enters hepatocytes via the liver-specific transporter OATP1B1. Heterodimerizes with the nuclear receptor RXR and binds to gene regulatory regions, modulating the transcription of various metabolic genes. Effects include upregulating genes related to mitochondrial biogenesis, mitophagy, fatty acid β-oxidation, and lipophagy, while downregulating genes involved in lipid droplet assembly, thereby reducing hepatic lipotoxicity	Indirect	Mitochondria (fatty acid oxidation, oxidative phosphorylation, biogenesis, autophagy); Lipid Droplet (lipid assembly, lipophagy); Lysosome (autophagy)
Pioglitazone	Phase IV	PPARγ agonist. Primarily acts on adipocytes, promoting adipocyte differentiation and increasing lipid storage capacity, thereby reducing free fatty acid flux to the liver. This action improves insulin sensitivity and effectively alleviates hepatic steatosis. Some studies show it can also inhibit the activation of hepatic stellate cells (HSCs), reducing fibrosis	Indirect	Lipid Droplet (lipid storage); Indirectly affects mitochondrial function
Berberine	Phase II	Indirect AMPK activator. Primarily activates AMPK by inhibiting mitochondrial respiratory chain complex I. Activated AMPK phosphorylates and inhibits acetyl-CoA carboxylase (ACC), reducing malonyl-CoA synthesis and thus relieving inhibition of mitochondrial fatty acid oxidation. It also initiates PGC-1α-mediated mitochondrial biogenesis and ULK1-mediated autophagy pathways	Indirect	Mitochondria (fatty acid oxidation, biogenesis); Lysosome (autophagy)
Survodutide	Phase III	Glucagon/GLP-1 dual agonist. Combines the satiety-promoting effect of GLP-1 with the energy expenditure-increasing effect of glucagon. Acts directly on hepatocytes to promote fatty acid β-oxidation and activates thermogenesis in brown adipose tissue (BAT), effectively reducing fat content in adipose tissue and liver	Indirect	Mitochondria (fatty acid oxidation, energy expenditure); Lipid Droplet (thermogenesis in adipose tissue)
Fenofibrate	Phase II	PPARα agonist. Binds to and activates this nuclear receptor, regulating the transcription of downstream genes. Effects include enhancing fatty acid oxidation in mitochondria and peroxisomes. It also increases the number and enzyme activity of hepatic peroxisomes, significantly promoting β-oxidation of very long-chain and branched-chain fatty acids	Indirect	Peroxisome (number, enzyme activity, β-oxidation); Mitochondria (fatty acid oxidation)
CRMP (Liver-targeted DNP prodrug)	Preclinical	Liver-targeted mitochondrial uncoupler. Eliminates the proton gradient across the mitochondrial inner membrane, dissipating energy from the electron transport chain as heat, thereby increasing energy expenditure and promoting lipid breakdown	Direct	Mitochondria (uncoupling)
Niclosamide ethanolamine	Preclinical	Mitochondrial uncoupler with a dual mechanism involving Nrf2 signaling pathway activation. Its uncoupling effect reduces mitochondrial ROS, while Nrf2 activation promotes cytoprotective and antioxidant gene expression, working together to reduce lipotoxicity	Direct	Mitochondria (uncoupling)
BAM15	Preclinical	Novel mitochondrial uncoupler. Acts as a non-protonophore, uncoupling the electron transport chain from ATP synthesis by increasing the permeability of the mitochondrial inner membrane, increasing energy expenditure, and potentially reducing ROS production	Direct	Mitochondria (uncoupling)
MSDC-0602K	Phase III	Mitochondrial pyruvate carrier (MPC) inhibitor. By blocking pyruvate entry into mitochondria, it initiates metabolic crosstalk, stimulating the catabolism of branched-chain amino acids (BCAAs), leading to improved insulin sensitivity	Direct	Mitochondria (metabolic substrate transport)
Urolithin A	Preclinical	Protects hepatocytes and alleviates steatosis; improves calcium homeostasis by modulating SERCA on MAMs, reducing ER stress	Indirect	Mitochondria-Associated Membranes (MAMs)
Statins	Phase IV	Reduce blood lipids and intrahepatic triglyceride content; inhibit SREBP2 to reduce PLIN5 expression, promoting lipolysis	Indirect	Mitochondria-Lipid Droplet contact
JTTZF (Chinese Herbal Formula)	Preclinical	Improves hepatic steatosis and oxidative stress; affects fatty acid metabolic reprogramming mediated by the PEX2/ATGL axis by downregulating ABCD2	Indirect	Peroxisome-Lipid Droplet contact site
ION224	Phase II	Significantly improves liver steatosis and fibrosis; specifically targets and inhibits DGAT2, inhibiting triglyceride synthesis at the ER-Lipid Droplet contact site and feedback regulating SREBP-1c via PE levels	Direct	Endoplasmic Reticulum-Lipid Droplet contact site (ER-LD)
DA-1241	Preclinical	GPR119 agonist. Induces nuclear translocation of transcription factor EB (TFEB) in an AMPK-dependent manner, upregulating autophagy and lysosome-related gene expression, enhancing lipophagy	Indirect	Lysosome, Lipid Droplet (lipophagy)
Tetrahydrocurcumin (THC)	Preclinical	Inhibits the mTORC1 signaling pathway, relieving its inhibition on TFEB, promoting its nuclear translocation, thereby globally upregulating autophagic flux and lysosome biogenesis, restoring lipophagy	Indirect	Lysosome, Lipid Droplet (lipophagy)
Trehalose	Preclinical	Natural disaccharide. Modulates the IRE1α-TFEB signaling pathway through interaction with IRE1α and TFEB, alleviating ER stress while enhancing autophagy-lysosome function	Indirect	Endoplasmic Reticulum (UPR), Lysosome (autophagy)
Chondroitin Sulfate (CS)	Preclinical	Restores lysosomal acidification and function, promotes lysosome-lipid droplet fusion (lipophagy) and drives mitochondrial fission to facilitate mitophagy	Direct	Lysosome, Lipid Droplet (lipophagy), Mitochondria (mitophagy)
Dual-targeted siRubicon NPs	Preclinical	Oxidative stress-responsive and liver-targeting nanoparticles delivering siRNA to silence the autophagy negative regulator Rubicon, specifically triggering hepatocyte lipophagy	Indirect	Lysosome, Lipid Droplet (lipophagy)
CsA@Dex-Gal/TPP NPs	Preclinical	Programmable organelle-targeting nanoparticles. Escape the acidic lysosomal environment first, then target mitochondria, releasing Cyclosporin A (CsA) *in situ* to inhibit mPTP opening, restore mitochondrial function, and promote mitophagy	Direct	Mitochondria (membrane potential), Lysosome (mitophagy)
Buddleoside	Preclinical	Natural flavonoid compound. Directly binds to and activates AMPK, subsequently inhibiting mTORC1 activity, driving the transcription factor EB (TFEB)-mediated autophagy-lysosome gene transcription program, alleviating steatohepatitis	Indirect	Lysosome (autophagy)
DWN12088	Preclinical	PRS inhibitor. Alleviates ER stress (ERS)-induced hepatocyte lipoapoptosis and inflammation by inhibiting the PERK/eIF2α/ATF4/CHOP signaling pathway	Indirect	Endoplasmic Reticulum (UPR)
Vitamin E	Phase III	Classic fat-soluble antioxidant. Directly incorporates into biological membranes (e.g., mitochondrial membrane, ER membrane), neutralizes lipid peroxidation radicals, blocks the chain reaction of lipid peroxidation, and protects membrane structural integrity	Direct	Membrane systems (Mitochondria, Endoplasmic Reticulum, etc. membranes)
AntiOxCIN4	Preclinical	Mitochondria-targeted antioxidant. The TPP^+^ moiety drives efficient enrichment within the mitochondrial matrix; the caffeic acid moiety directly scavenges ROS and upregulates antioxidant defense by activating the Nrf2/Keap1 and PGC-1α/SIRT3 axes	Direct	Mitochondria (antioxidant)
Curcumin	Phase III	Multi-target modulator. Directly scavenges ROS; inhibits the NF-κB pathway to reduce inflammation; activates the Nrf2 pathway to enhance antioxidant enzyme expression; also improves insulin signaling pathways	Indirect	Mitochondria (antioxidant, improves function)

Abbreviations: ACC, Acetyl-CoA carboxylase; AMPK, AMP-activated Protein Kinase; ATF4, Activating Transcription Factor 4; BAT, brown adipose tissue; BCAA, Branched-Chain Amino Acid; CHOP, C/EBP, homologous protein; CRMP, Controlled-release mitochondrial protonophore; CsA, Cyclosporin A; DGAT2, Diacylglycerol O-Acyltransferase 2; DNP, 2,4-Dinitrophenol; eIF2α, Eukaryotic Initiation Factor 2 Alpha; ER, endoplasmic reticulum; ER-LD, Endoplasmic Reticulum-Lipid Droplet; ERS, ER, stress; FDA, food and drug administration; GLP-1, Glucagon-like Peptide-1; GPR119, G Protein-Coupled Receptor 119; HSC, hepatic stellate cell; IRE1α, Inositol-requiring Enzyme 1 Alpha; MAM, Mitochondria-Associated Membrane; MASH, Metabolic Dysfunction-associated Steatohepatitis; mPTP, mitochondrial permeability transition pore; MPC, mitochondrial pyruvate carrier; mTORC1, Mechanistic Target of Rapamycin Complex 1; NF-κB, Nuclear Factor Kappa-Light-Chain-Enhancer of Activated B Cells; Nrf2, Nuclear Factor Erythroid 2-Related Factor 2; OATP1B1, Organic Anion Transporting Polypeptide 1B1; PC, phosphatidylcholine; PE, phosphatidylethanolamine; PERK, PKR-like ER, kinase; PEX2, Peroxisomal Biogenesis Factor 2; PGC-1α, Peroxisome Proliferator-activated Receptor Gamma Coactivator 1-alpha; PLIN5, Perilipin 5; PPARα, Peroxisome Proliferator-activated Receptor Alpha; PPARγ, Peroxisome Proliferator-activated Receptor Gamma; PRS, Prolyl-tRNA, synthetase; ROS, reactive oxygen species; RXR, Retinoid X Receptor; SERCA, Sarco/Endoplasmic Reticulum Ca^2+^ ATPase; siRNA, Small Interfering RNA; SIRT3, Sirtuin 3; SREBP-1c, Sterol Regulatory Element-Binding Protein 1c; SREBP2, Sterol Regulatory Element-Binding Protein 2; TFEB, Transcription Factor EB; THC, tetrahydrocurcumin; THR-β, thyroid hormone receptor beta; TPP^+^, triphenylphosphonium; ULK1, Unc-51, Like Autophagy Activating Kinase 1; UPR, unfolded protein response.

**FIGURE 3:**
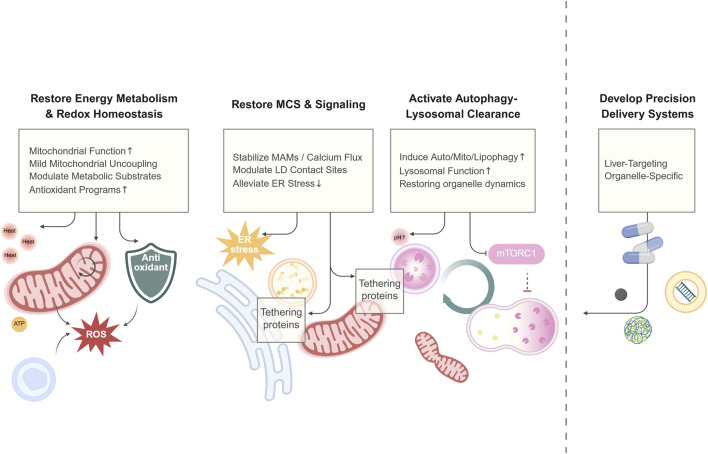
A conceptual framework of therapeutic strategies for MASLD. Emerging therapies for MASLD move beyond macroscopic metabolic regulation to directly target the subcellular dysfunction driving disease progression. This framework organizes these strategies into four foundational pillars: Energetic and Redox Repair (e.g., enhancing mitochondrial function and antioxidant defenses), Organelle Connectivity and Signaling (e.g., stabilizing MCSs to ameliorate stress), Clearance and Renewal (e.g., boosting autophagic flux), and Precision Targeting (e.g., using nanocarriers for organelle-specific delivery). This integrated approach aims to restore metabolic flexibility by reconstituting organellar homeostasis. Created in BioRender.

The essence of aberrant hepatic metabolism is the declined adaptive capacity of the organism to nutritional changes, i.e., metabolic inflexibility, which relies on the coordinated interplay of multiple organelles at the subcellular level. During the progression of MASLD, key organelles in hepatocytes all show functional abnormalities and structural disorganization. These abnormalities cover multiple key pathological links, including energy metabolism imbalance, exacerbated oxidative stress, autophagy impairment, and biogenesis and material transport disorders. More importantly, the functional dysregulation of MCSs between these organelles further disrupts substance exchange and signal transduction between organelles, exacerbating lipid deposition, lipotoxicity response, and cell damage, and ultimately driving disease progression. Emphasizing organelle mechanisms is to build a bridge between “efficacy and target”: mitochondria, the ER, LDs, and their membrane contact network are hubs for lipid metabolism, oxidative stress, and response regulation. Understanding these mechanisms will facilitate efficacy prediction, precise patient subtyping, the design of combination therapies, and the identification of novel therapeutic windows (Figure 3).

### 4.1 Energy metabolism regulation

Mitochondria are central to fatty acid oxidation, ATP production, and metabolic signal integration. The strategy of targeting mitochondrial function has expanded from simply improving oxidative phosphorylation efficiency to also enhancing dynamic regulation and substrate availability.

THR-β agonists, such as resmetirom, function by activating the nuclear receptor THR-β within hepatocytes, thereby regulating the transcription of associated genes. This transcriptional modulation enhances both mitochondrial fatty acid oxidation and mitochondrial biogenesis, which collectively alleviate cellular energy stress (Brisnovali et al., 2024). AMPK agonists, such as Metformin and Berberine, serve as indirect activators of the energy sensor AMPK. Upon activation, AMPK phosphorylates and inhibits acetyl-CoA carboxylase (ACC), which in turn reduces the synthesis of malonyl-CoA, thereby lifting the inhibitory brake on mitochondrial fatty acid oxidation. This process is complemented by the activation of PGC-1α-mediated mitochondrial biogenesis (N. Li et al., 2025). GLP-1/GIP dual agonists, such as Survodutide, exert their pleiotropic effects through complex signaling cascades. While reducing caloric intake, these agents also promote fatty acid β-oxidation and increase resting energy expenditure (Romero-Gómez et al., 2023). PPAR agonists, including Fenofibrate and Lanifibranor, act by binding to and activating their respective nuclear receptors. This activation of downstream gene transcription enhances fatty acid oxidation within both hepatic mitochondria and peroxisomes (Gawrieh et al., 2021). The above drugs, whether by regulating nuclear receptor gene expression or through signaling cascades, have pleiotropic and synergistic effects and also show indirect regulatory effects on organelles and their interactions.

A classic drug that directly targets mitochondria is the uncoupler, which blocks ADP phosphorylation by eliminating the proton gradient in the mitochondrial inner membrane, causing the energy from the electron transport chain to be released as heat. The classic 2,4-dinitrophenol (DNP) has been limited in clinical use due to its high systemic toxicity. To overcome this challenge, investigators have developed liver-targeted, controlled-release DNP analogues aimed at mitigating systemic toxicity and achieving localized hepatic energy expenditure. Among these, a controlled-release mitochondrial protonophore (CRMP) demonstrated significant therapeutic efficacy in animal models. It safely reversed hypertriglyceridemia and hepatic steatosis in non-human primate models, and in aged mice fed a HFD, it safely reduced hepatic lipid content and insulin resistance, while also ameliorating hepatic oxidative stress and inflammation, and extending their lifespan (Goedeke et al., 2022; Goedeke et al., 2019). Niclosamide, an oral anthelmintic drug, operates through a dual mechanism of mitochondrial uncoupling and Nrf2 signaling pathway activation, which enables it to reverse HFD-induced hyperglycemia and fatty liver in animal models (Park et al., 2019). Another mitochondrial uncoupler, BAM15, is a non-protonophore with a superior safety profile. Head-to-head comparisons have shown it to be more effective than niclosamide in improving metabolic parameters (Chen et al., 2024). Furthermore, a synergistic effect was observed when BAM15 was combined with the THR-β agonist resmetirom, which significantly reduced hepatic steatosis and improved metabolism in a mouse model of fatty liver disease (Zhou et al., 2024).

In addition, some drugs directly act on specific transporters on the mitochondrial membrane to regulate the supply of metabolic substrates and energy metabolism within mitochondria. For instance, the mitochondrial pyruvate carrier (MPC) inhibitor, MSDC-0602K, initiates metabolic crosstalk by blocking the entry of pyruvate into mitochondria, thereby stimulating branched-chain amino acid (BCAA) catabolism. This mechanism has demonstrated therapeutic efficacy in clinical trials, showing an improvement in insulin resistance (Ferguson et al., 2023; Harrison et al., 2020a).

### 4.2 Autophagy-clearance pathway activation

The clearance capacity for damaged organelles and excess LDs is crucial for maintaining hepatocyte homeostasis. In recent years, drug research targeting the autophagy-lysosome pathway has advanced rapidly, benefiting from new technologies such as nanotechnology, RNA interference, and high-throughput compound screening.

Inhibiting the autophagy negative regulator mTORC1 represents a classic strategy. Tetrahydrocurcumin (THC) promotes TFEB activation and lipophagy through this mechanism (Wu et al., 2024). The natural flavonoid compound buddleoside activates AMPK, thereby inhibiting mTORC1 and activating TFEB (Chen et al., 2025). Another flavonoid, quercetin, can upregulate autophagy, although its specific mechanism remains unclear (Katsaros et al., 2024). Hesperetin indirectly inhibits mTORC1 by suppressing glutaminolysis, while licochalcone A promotes autophagy by inhibiting mTOR and upregulating the activity of the ULK1/Beclin-1/VPS34 complex (J. Li et al., 2025; W. Li et al., 2025).

TFEB is a core transcription factor regulating autophagy. The GPR119 agonist DA-1241 induces TFEB nuclear translocation, upregulates genes related to autophagy and lysosome biogenesis, increases lysosomal number and activity, enhances lipophagy, and reduces hepatic lipid levels (Yoo et al., 2025). High-throughput screening has also identified various natural TFEB agonists, such as tanshinone IIA (Zheng et al., 2024). The natural disaccharide trehalose can bind both IRE1α and TFEB, alleviating ER stress while simultaneously enhancing autophagy (Su et al., 2025). Furthermore, the antihistamine drug desloratadine has been identified as an AMPK-dependent TFEB agonist (Lin et al., 2024). All these compounds have demonstrated efficacy in ameliorating hepatic steatosis in animal models.

Restoring lysosomal acidification function constitutes another strategy. The natural nanomaterial attapulgite (ATT) can be internalized into lysosomes and effectively restore their acidification status (Hao et al., 2025). Chondroitin sulfate (CS), while restoring lysosomal acidification, can also drive mitochondrial fission and mitophagy (Sun et al., 2025).

Other therapeutic agents that enhance mitophagy include monobutyrin (MB) and steam explosion-modified pea peptides (SE-modified PP), which also possess metabolic pathway regulatory effects (Zhang et al., 2025; Zhang et al., 2025). The novel small molecule TJ0113 induces mitophagy by activating the PINK1/Parkin pathway, ameliorating lipid accumulation, inflammation, and fibrosis (Huang et al., 2025).

Leveraging advanced delivery technologies, hepatocyte-targeting siRNA nanoliposomes (Glipo-siRubi) effectively ameliorate lipid deposition and ER stress by efficiently silencing the autophagy negative regulator Rubicon (Zhang et al., 2025). A recently developed dual-targeted (oxidative stress-responsive and liver-targeting) nanoparticle system further potently triggers hepatocellular lipophagy through the delivery of siRubicon, demonstrating the considerable potential of RNAi therapy (Lan et al., 2025). Moreover, more precise organelle-specific strategies, such as mitochondria-targeted nanoparticles (CsA@Dex-Gal/TPP), can accurately deliver cyclosporine A to inhibit mitochondrial permeability transition pore (mPTP) opening, thereby restoring mitochondrial function and mitophagy (Zhang et al., 2025).

### 4.3 Oxidative stress intervention

In MASLD, ER stress and oxidative stress form a vicious cycle. To alleviate ER stress, traditional Chinese medicine formulations (e.g., Qinlian Hongqu decoction and the ZhiMu-HuangBai herb pair) suppress lipid synthesis by inhibiting the IRE1α–XBP1s pathway, whereas the prolyl-tRNA synthetase inhibitor attenuates ER stress and hepatic steatosis by blocking the PERK–eIF2α–ATF4 pathway (Lee et al., 2024; Y. Zhang et al., 2024; Zhao et al., 2024). Regarding antioxidant therapy, coenzyme Q10, vitamin E, and curcumin have shown clinical efficacy in randomized controlled trials (RCTs) in MASLD patients (Song et al., 2025; Vrentzos et al., 2024; Yaikwawong et al., 2025). In addition, compounds such as caffeic acid and asiaticoside activate Nrf2, thereby enhancing the activity of antioxidant enzymes and reducing lipotoxicity and oxidative damage (Y. Wei et al., 2025; J. Zhang et al., 2024). Both the mitochondria-targeted compound AntiOxCIN4 and the mitochondrial metabolite α-ketoglutarate (AKG) activate the Nrf2 pathway, leading to a significant improvement in mitochondrial function and an enhancement of the antioxidant defense system (Amorim et al., 2022; Cheng et al., 2024). All these agents have shown efficacy in reducing lipid accumulation and liver injury in MASLD animal models.

Nanotechnology-based strategies have further improved targeting specificity and antioxidant efficacy, as exemplified by liver-targeted selenium nanoparticles (GA-MSe), intestine–liver dual-targeted nanoparticles (AXT@TWG@LBGs), ROS-responsive nanobubbles (Apt-DTP-NBs@RSV@OCA) for controlled drug release, and MXene nanozymes (Nb_4_C_3_/Ta_4_C_3_) (S. He et al., 2025b; 2025a; Lei et al., 2025; Lin et al., 2025; Zhang et al., 2025).

### 4.4 Membrane contact repair

During MASLD progression, the dysfunction of MCSs exacerbates organelle decoupling, leading to metabolic and quality control dysregulation. Consequently, targeting these MCSs is emerging as a novel therapeutic strategy.

Some drugs and therapies do not directly act on MCSs tethering proteins but can exert beneficial indirect effects on MCSs’ dynamics and function. Natural molecules like Urolithin A improve calcium homeostasis by acting on SERCA within MAMs, indirectly modulating MAMs calcium signaling and alleviating palmitic acid-induced ER stress (Ryu et al., 2024). Statins, on the other hand, can decrease PLIN5 expression by inhibiting SREBP2, thereby affecting mitochondria-lipid droplet contact and promoting lipolysis (Langhi et al., 2014). Non-pharmacological interventions, such as photobiomodulation and exercise, also improve MAMs contact and PDM function by upregulating MFN2 (Bórquez et al., 2024; Jiang et al., 2025). Moreover, studies have found that the traditional Chinese medicine formula Jiangtang Tiaozhi Formula (JTTZF) indirectly regulates the Pex-LD interface by downregulating ABCD2, which affects the PEX2/ATGL axis (Miao et al., 2025).

Among direct targeting strategies, the antisense oligonucleotide DGAT2 inhibitor, ION224, inhibits lipid synthesis at the ER-lipid droplet contact sites and feedback-regulates SREBP-1c. It has shown promising results in clinical trials by improving both steatosis and fibrosis (Loomba et al., 2025). While modulators targeting MFN2 and Vdac are mostly in the preclinical stage, direct interventions on MCSs show great potential (Zhou et al., 2025). However, most strategies, including DGAT2 inhibition, still face multiple challenges due to target complexity and clinical translation.

## 5 Discussion

In conclusion, MASLD is a complex and heterogeneous metabolic disorder, with its pathological mechanism rooted not only in the dysfunction of individual organelles but also in the rigidity and interruption of their interactive networks. This review highlights how mitochondria, the ER, LDs, autophagic pathways, and their MCSs collaboratively regulate lipid metabolism, energy homeostasis, oxidative stress, and inflammatory responses.

Currently, while many drugs targeting macroscopic metabolic pathways have shown promise in the clinic, their application for the precise treatment of MASLD subtypes, especially in lean or non-diabetic patient populations, remains limited. Therefore, we believe that therapies precisely targeting organelles and their interactions have the potential to improve MASLD pathophysiology by restoring cellular metabolic flexibility through the specific repair of MCSs, the activation of selective autophagic pathways, and the re-engagement of key energy metabolic pathways. However, the translation of this strategy into clinical application still faces challenges, including how to achieve precise subcellular delivery and how to dynamically monitor efficacy through non-invasive methods.

Looking to the future, we anticipate that the integration of cross-disciplinary technologies, such as organelle-targeted nano-delivery systems and cell imaging techniques to validate inter-organelle dynamics, will provide technical support and pave the way for the realization of “precision organelle medicine” and offer more targeted and safer treatment options for MASLD.

## Glossary

ACC: Acetyl-CoA carboxylase
AKG: α-Ketoglutarate
AMPK: AMP-activated protein kinase
ATF4/6: Activating transcription factor 4/6
ATGL: Adipose triglyceride lipase
BCAA: Branched-chain amino acid
BMI: Body mass index
CAT: Catalase
CCR: C-C chemokine receptor type
CE: Cholesterol ester
CGI-58: Comparative gene identification-58
CMA: Chaperone-mediated autophagy
CM: Cytoplasmic mitochondria
CRMP: Controlled-release mitochondrial protonophore
CYP: Cytochrome P450
DAMP: Danger-associated molecular pattern
DNL: *De novo* lipogenesis
DNP: 2,4-Dinitrophenol
DRP1: Dynamin-related protein 1
eIF: Eukaryotic initiation factor
ER: Endoplasmic reticulum
ERAD: ER-associated degradation
ETC: Electron transport chain
FAO: Fatty acid oxidation
FASN: Fatty acid synthase
FGF21: Fibroblast growth factor 21
GLP-1: Glucagon-like peptide-1
GP73: Golgi protein 73
GSH: Glutathione
HCC: Hepatocellular carcinoma
HFD: High-fat diet
HSC: Hepatic stellate cell
IP3R: Inositol 1,4,5-trisphosphate receptor
IRE1α: Inositol-requiring enzyme 1α
LD: Lipid droplet
MAM: Mitochondria-associated ER membrane
MASLD: Metabolic dysfunction-associated steatotic liver disease
MAFLD: Metabolic dysfunction-associated fatty liver disease
MCS: Membrane contact site
MERC: Mitochondria-ER contact
MFN1/2: Mitofusin 1/2
MPC: Mitochondrial pyruvate carrier
mPTP: Mitochondrial permeability transition pore
mTORC1: Mechanistic target of rapamycin complex 1
mtDNA: Mitochondrial DNA
NAFLD: Non-alcoholic fatty liver disease
NASH: Non-alcoholic steatohepatitis
NOX: NADPH oxidase
Nrf2: Nuclear factor erythroid 2-related factor 2
OPA1: Optic atrophy 1
PDM: Peridroplet mitochondria
PERK: PKR-like ER kinase
PGC-1α: Peroxisome proliferator-activated receptor gamma coactivator 1-alpha
PPAR: Peroxisome proliferator-activated receptor
ROS: Reactive oxygen species
RyR: Ryanodine receptor
SERCA: Sarco/endoplasmic reticulum Ca2+-ATPase
SOD: Superoxide dismutase
SREBP: Sterol regulatory element-binding protein
TAG: Triacylglycerol (Triglyceride)
TCA cycle: Tricarboxylic acid cycle (Krebs cycle)
TFAM: Mitochondrial transcription factor A
TFEB: Transcription factor EB
THR-β: Thyroid hormone receptor beta
UPR: Unfolded protein response
VLDL: Very-low-density lipoprotein
VLCFA: Very-long-chain fatty acid
